# BRAIN 2.0: Time and Memory Complexity Improvements in the Algorithm for Calculating the Isotope Distribution

**DOI:** 10.1007/s13361-013-0796-5

**Published:** 2014-02-12

**Authors:** Piotr Dittwald, Dirk Valkenborg

**Affiliations:** 1 College of Inter-faculty Individual Studies in Mathematics and Natural Sciences, University of Warsaw, Warsaw, Poland; 2 Institute of Informatics, University of Warsaw, Warsaw, Poland; 3 Applied Bio and Molecular Systems, VITO, Mol, Belgium; 4 Interuniversity Institute for Biostatistics and Statistical Bioinformatics, Hasselt University, Diepenbeek, Belgium; 5 Center for Proteomics, Antwerp, Belgium

**Keywords:** Isotopic distribution, Isotopic abundance’s ratios, Mass spectrometry, Proteomics, BRAIN algorithm

## Abstract

Recently, an elegant iterative algorithm called BRAIN (**B**affling **R**ecursive **A**lgorithm for **I**sotopic distributio**N** calculations) was presented. The algorithm is based on the classic polynomial method for calculating aggregated isotope distributions, and it introduces algebraic identities using Newton-Girard and Viète’s formulae to solve the problem of polynomial expansion. Due to the iterative nature of the BRAIN method, it is a requirement that the calculations start from the lightest isotope variant. As such, the complexity of BRAIN scales quadratically with the mass of the putative molecule, since it depends on the number of aggregated peaks that need to be calculated. In this manuscript, we suggest two improvements of the algorithm to decrease both time and memory complexity in obtaining the aggregated isotope distribution. We also illustrate a concept to represent the element isotope distribution in a generic manner. This representation allows for omitting the root calculation of the element polynomial required in the original BRAIN method. A generic formulation for the roots is of special interest for higher order element polynomials such that root finding algorithms and its inaccuracies can be avoided.

**Electronic supplementary material:**

The online version of this article (doi:10.1007/s13361-013-0796-5) contains supplementary material, which is available to authorized users.

**Graphical abstract:**
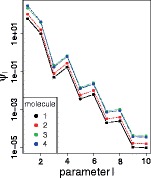
ᅟ

## Introduction

During the last decade there seems to be a revived interest in methods that calculate the isotope distribution of molecules when the molecular formula is given. Numerous publications and discussions in the literature do witness this trend [1-7]. A recent review article by Valkenborg et al. [8] gives an extensive overview of the methodology. However, Claesen et al. [9] introduced a new method called BRAIN (**B**affling **R**ecursive **A**lgorithm for **I**sotopic distributio**N** calculations) that is able to compute the aggregated isotope distributions and their corresponding center-masses. The BRAIN method is based on the polynomial expansion of the element polynomials as described by [10, 11]. Of note, instead of expanding the polynomial using a symbolic approach [12-15], fast Fourier transform approach (FFT) [16-20], or just explicitly perform the polynomial multiplications [21, 22], the BRAIN method employs an iterative algorithm that exploits the algebraic identities of polynomial power series. For this purpose, BRAIN uses two polynomial generating functions that rely on the theory of Newton-Girard and Viète’s formulae. These two generating functions calculate the aggregated distribution and corresponding center-masses. Interestingly, the generating function approach for center-masses was also used by Fernandez-de-Cossio Diaz and Fernandez-de-Cossio and implemented in a software using the FFT-approach [6].

The advantage of BRAIN lies in its simple implementation and has been shown to be as accurate as existing methods: Emass and SIRIUS [4, 5, 7, 9, 23, 24]. However, it has been found by [6] that the computational complexity is asymptotically suboptimal in comparison with their fast Fourier-based algorithm. A first reason for this suboptimal behavior is that BRAIN requires starting the iteration at the lightest variant because of the nature of Newton-Girard’s identities [25]. In theory, one can start the iteration from the heaviest variant, but this non-standard use of the algorithm will not be discussed here. Second, for each aggregated isotope variant, two additional terms are stored in the memory for further usage during the iterative process. Previous properties result in a BRAIN algorithm that has a computational complexity of order *O*(*N* ^2^), as described by [6].

In this paper, we introduce two improvements to the original BRAIN method that optimize the algorithm in terms of memory and time complexity without compromising its simplicity of implementation as an iterative algorithm. The gain in efficiency is especially noticeable when calculating large molecules (e.g., 50 or more aggregated isotope variants to adequately span the isotope distribution). As such, for small molecules, we suggest to revert to the original BRAIN method [7]. It should be noted that the presented improvements are only intended for the calculation of the aggregated isotope distribution and not for the center-masses. Currently, we are investigating whether the improvements are also suitable for the center-mass calculation.

Furthermore, we introduce a new formulation to represent element polynomials in a generic form. Doing so, we avoid the calculation of the roots of the element polynomial, which are required in the original BRAIN approach. This third improvement is especially interesting when the molecular formula includes elements with many isotopes (e.g., platinum). Such a poly-isotopic element will result in a high-order element polynomial for which the roots cannot be calculated explicitly or are complicated to compute.

All proposed improvements are based on mathematical concepts that simplify the original BRAIN approach. We will provide an intuitive reasoning for each of these improvements. Since the BRAIN method has already been extensively validated in the literature, we will compare the impact of the improvements only to the original algorithm.

## Methods

Before going into detail about the three improvements, we provide the basic concepts of the original BRAIN method. The overview is provided in the section about the standard BRAIN algorithm. The section about BRAIN 2.0 deals with the proposed improvements.

### Standard BRAIN Algorithm

Consider a molecule composed of *v* carbon, *w* hydrogen, *x* nitrogen, *y* oxygen, and *z* sulphur atoms (i.e., having chemical formula *C*_*v*_ *H*_*w*_ *N*_*x*_ *O*_*y*_ *S*_*z*_). Such a molecule can be represented by a polynomial generating function:

$$ \begin{array}{lll}Q\left(I;v,w,x,y,z\right)\hfill & =\hfill & {\displaystyle {\left({P}_{C_{12}}{I}^0+{P}_{C_{13}}{I}^1\right)}^v}\times {\displaystyle {\left({P}_{H_1}{I}^0+{P}_{H_2}{I}^1\right)}^w}\hfill \\ {}\hfill & \times \hfill & {\displaystyle {\left({P}_{N_{14}}{I}^0+{P}_{N_{15}}{I}^1\right)}^x}\times {\displaystyle {\left({P}_{O_{16}}{I}^0+{P}_{O_{17}}{I}^1+{P}_{O_{18}}{I}^2\right)}^y}\hfill \\ {}\hfill & \times \hfill & {\displaystyle {\left({P}_{S_{32}}{I}^0+{P}_{S_{33}}{I}^1+{P}_{S_{34}}{I}^2+{P}_{S_{36}}{I}^4\right)}^z}\hfill \\ {}\hfill & =\hfill & {\left\{{Q}_C(I)\right\}}^v\times {\left\{{Q}_H(I)\right\}}^w\times {\left\{{Q}_N(I)\right\}}^x\times {\left\{{Q}_O(I)\right\}}^y\times {\left\{{Q}_S(I)\right\}}^z.\hfill \end{array} $$

The polynomial generating function is composed of a multiplication of simple element polynomials *Q*_*C*_(*I*), *Q*_*H*_(*I*), …, *Q*_*S*_(*I*), which are raised to a power that corresponds to the number of elements in the molecule. An important variable in this polynomial is the indicator variable *I*, whereas its power denotes the additional neutrons compared with the lightest variant, or the monoisotopic variant in the case of C, H, N, O, and S. The coefficients ${P}_{C_{12}}$, ${P}_{C_{13}}$, …, ${P}_{S_{36}}$ are the probabilities of occurrence related to the stable isotopes of previous elements. In order to obtain the aggregated isotope distribution, the expansion of the polynomial *Q* is of interest:

1 $$ Q\left(I;v,w,x,y,z\right)={\displaystyle \sum_{j=0}^n}\;{q}_j{I}^j $$

where *n* = *v* + *w* + *x* + 2*y* + 4*z* indicates the order of the expanded polynomial. The coefficients *q*_*j*_ are meaningful as they correspond to the probability of *j*-th aggregated isotope variant (i.e., the molecule with *j* additional neutrons compared to the monoisotopic one). BRAIN adopts an iterative scheme that calculates *q*_*j*_ as a function of its lighter aggregated isotope variants that are calculated in a previous iteration of the procedure:

2 $$ {q}_j=-\frac{1}{j}{\displaystyle \sum_{l=1}^j}\;{q}_{j-l}{\psi}_l $$

where *ψ*_*l*_ is a power sum of the roots of the element polynomials of *Q*_*C*_(*I*), *Q*_*H*_(*I*), …, *Q*_*S*_(*I*). The term *ψ*_*l*_ can be calculated as follows:

3 $$ {\psi}_l=v{\left({r}_C\right)}^{-l}+w{\left({r}_H\right)}^{-l}+x{\left({r}_N\right)}^{-l}+y\left({r}_{O, all,l}\right)+z\left({r}_{S, all,l}\right) $$

where *r*_*C*_, *r*_*H*_, *r*_*N*_ are the roots of the element polynomials *Q*_*C*_(*I*), *Q*_*H*_(*I*) and *Q*_*N*_(*I*). For simplicity, oxygen and sulphur, which have more than one root, will be denoted by the notation ${r}_{O, all,l}={\left({r}_O\right)}^{-l}+{\left({\displaystyle {\overline{r}}_O}\right)}^{-l}$ and ${r}_{S, all,l}={\left({r}_{S,1}\right)}^{-l}+{\left({\displaystyle {\overline{r}}_{S,1}}\right)}^{-l}+{\left({r}_{S,2}\right)}^{-l}+{\left({\displaystyle {\overline{r}}_{S,2}}\right)}^{-l}$. The roots can be pre-computed as a closed form equation or derived by numerical root finding methods. Typically, the iteration is started from the lightest isotope variant. However, duality in the Newton-Girard formulae allows it to start from the heaviest isotope variant as well. More details about the BRAIN algorithm can be found in the presentation of Claesen et al. [9].

### BRAIN 2.0

BRAIN 2.0 includes two improvements that reduce the complexity of the computation. The first improvement reduces the length of the summation in Equation (2) for accurately calculating the isotope variant *q*_*j*_. The second improvement allows for a user-defined starting peak in the recursive procedure. Both steps lead to less demanding memory requirements and to a gain in computation time. The third improvement, a root omitting algorithm, is proposed to avoid the calculation of the roots of element polynomials used in *ψ*_*l*_. Instead, the sums of powered roots are represented as a function of the coefficients of the element polynomial by using the theorem of Newton-Girard. This representation allows for a generic form and implementation of the elements in BRAIN 2.0.

#### Recurrence of Constant Length [RCL]

**Figure 1:**
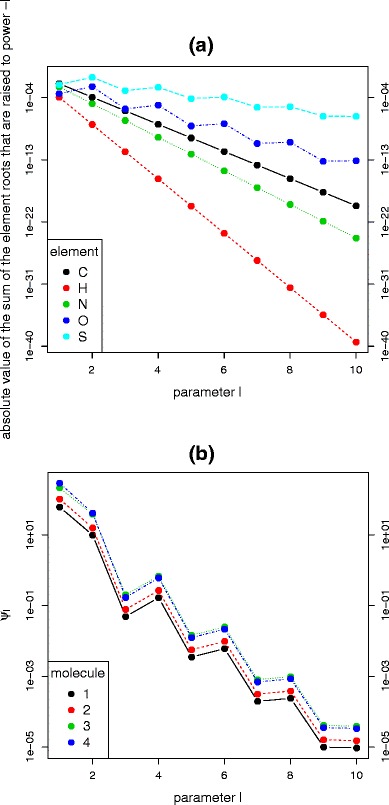
(**a**) For each atom we show the absolute value of the roots raised to the power − *l*, i.e., for carbon it is |(*r*_*C*_)^− *l*^| and for sulphur it is |*r*_*S*,*all*,*l*_| (the latter can be calculated using de Moivre’s formula or [RO] improvement pointed out in this manuscript). (**b**) For the four heaviest biomolecules from [26], we plot the absolute value of *ψ*_*l*_. Formulas and monoisotopic masses corresponding to the molecules are presented in Supplementary Table S1. Note that the scale of the y-axes in both panels is logarithmic

As we can observe in Equation (2), an aggregated isotope variant *q*_*j*_ is calculated based on the results from previous iterations. As a consequence, the calculation for coefficient *q*_*j*_ requires ${\scriptscriptstyle \frac{j\times \left(j+1\right)}{2}}$ multiplications. However, it should be noted that the multiplication involves the terms *q*_*j* − *l*_ and *ψ*_*l*_. The term *q*_*j* − *l*_ is a probability, which is by definition smaller than one. The term *ψ*_*l*_ is a power sum of the element roots, which becomes in general smaller for increasing *l*. This decreasing trend is caused by the high powers to which the roots are raised. Figure 1a illustrates how the power roots for carbon, hydrogen, nitrogen, oxygen, and sulphur decrease as a function of the power. Interestingly, the elements with two isotope variants decrease faster than the more complex elements (e.g., oxygen and sulphur).

Even for large molecules, for which the power roots are multiplied with large values for *v*, *w*, *x*, *y*, *z* as indicated in Equation (3), the term *ψ*_*l*_ will decrease to ignorable values at some point in the iteration. This principle is illustrated in Figure 1b for the four heavy biomolecules presented in the Supplementary Table S1. Note that these molecules correspond to molecules 7–10 used previously by Olson and Yergey [26], Claesen et al. [9], and Böcker [4] for the evaluation of NeutronCluster, BRAIN, and SIRIUS, respectively. The results in Figure 1 indicate that the summation in Equation (2) can be trimmed to a constant number of *d* iterations to ignore irrelevant values of *ψ*_*l*_. Obviously, this intervention is only valid when index *j* is larger than *d*. The summation from *l* = 1 to *j* in Equation (2) can be replaced by a summation to *d*:

4 $$ {q}_j=-\frac{1}{j}{\displaystyle \sum_{l=1}^d}\;{q}_{j-l}{\psi}_l\kern0.5em . $$

#### Late Starting Point [LSP]

As already pointed out by [6, 25] a limitation of the original BRAIN method is that the iterative procedure has to start from the lightest variant. This artefact is inconvenient when calculating very large molecules (cfr. human dynein heavy chain; *C*_23832_ *H*_37816_ *N*_6528_ *O*_7031_ *S*_170_) because the light isotope variants are not of interest as they often fall below the normal detection range of a mass spectrometer. The reason why the procedure has to start from the lightest or heaviest isotope variant is that the probability of occurrence has to be calculated exactly to receive the aggregated isotope distribution as a probability distribution and the information about previously calculated aggregated isotope variant is required to accurately calculate a new variant. However, when probabilities are not required, the relative isotope distribution (e.g., maximum peak normalized to 100 %), can be computed from any starting point. This concept is realized by the fact that Equation (2) is a linear function of the recursion starting point. As a consequence, the iterative procedure is independent from the starting values in terms of the ratios of consecutive peaks. Therefore, the starting value can be arbitrarily set, e.g. to 1.

Let us assume we are interested in the isotope distribution from peak *n*_start_ to *n*_stop_, then

1. The recursion shall start at variant *n*_start_ − *b* with coefficient ${q}_{n_{\mathrm{start}}-b}=1$ because *b* burn-in steps are required to stably calculate the coefficients (see heuristic from formula 10 for exemplary values). The starting point *n*_start_ and stopping point *n*_stop_ are user-defined parameters;
2. The next values ${q}_{n_{\mathrm{start}}-b+1},\dots$ are calculated using Equation (2) or Equation (4);
3. The maximum peak is normalized to 1.

As we start from an arbitrary selected value, the burn-in period *b* is needed for recovering the real proportions between the consecutive peaks. It is crucial that the procedure converges before the calculation of the *n*_start_ variant because previous results are being propagated in this calculation. The late starting option allows us to focus the calculation on the prominent part of the aggregated isotope distribution, similar in spirit as heterodyning in FFT-based algorithms [6, 16-20].

#### Root Omitting [RO]

It is intuitional that Newton-Girard identities can be used to find the roots for the element polynomials as well. Doing so, for each chemical element one may obtain a formula for *ψ*_*l*_ as a function of the coefficients of the element polynomials *Q*_*C*_(*I*), …, *Q*_*S*_(*I*) avoiding the direct calculation of the roots. This generic representation can be useful for elements with a large number of stable isotope forms when roots cannot be obtained from closed formulae or when closed formed formulae are cumbersome to notate. As we already have shown in Equation (3), *ψ*_*l*_ is a standard inner product of

- vector (*v*,*w*,*x*,*y*,*z*) denoting the element composition of the molecule;
- vector ((*r*_*C*_)^− *l*^,(*r*_*H*_)^− *l*^,(*r*_*N*_)^− *l*^,(*r*_*O*,*all*,*l*_),(*r*_*S*,*all*,*l*_)) indicating the power sum of the element roots.

The vector in (a) is given by the chemical formula of the molecule, whereas the vector in (b) can be calculated from the element roots of *C*, *H*, *N*, *O*, *S*. Note that only the power *l* will change during the iterations. We will illustrate the principle of root omitting for sulphur, however, the concept is similar for all atoms. By applying the Newton-Girard theorem from Equation (2) directly on the element polynomial of sulphur *Q*_*S*_(*I*), we obtain a following system of equations:

5 $$ \begin{array}{c}{P}_{S_{33}}=-{P}_{S_{32}}{r}_{S, all,1}\\ {}{P}_{S_{34}}=-\frac{1}{2}\left({P}_{S_{33}}{r}_{S, all,1}+{P}_{S_{32}}{r}_{S, all,2}\right)\\ {}0={P}_{S_{35}}=-\frac{1}{3}\left({P}_{S_{34}}{r}_{S, all,1}+{P}_{S_{33}}{r}_{S, all,2}+{P}_{S_{32}}{r}_{S, all,3}\right)\\ {}{P}_{S_{36}}=-\frac{1}{4}\left({P}_{S_{34}}{r}_{S, all,2}+{P}_{S_{33}}{r}_{S, all,3}+{P}_{S_{32}}{r}_{S, all,4}\right)\end{array} $$

Note that the development of the set of equations also includes the non-existing (we consider here only stable isotopes) sulphur isotope ^35^ *S* for which we set the probability equal to zero. From the first line in (5), the value of (*r*_*S*,*all*,1_) can be easily calculated from the known isotope distribution as $-{\scriptscriptstyle \frac{P_{S_{33}}}{P_{S_{32}}}}$. Given the results of the first equation, the root from the second equation (*r*_*S*,*all*,*2*_) can be obtained and so on. This recurrence procedure enables us to calculate the powered root up to (*r*_*S*,*all*,*4*_). Next, the higher power roots can be calculated by extending the procedure to nonexisting sulphur isotopes for which the probability of occurrence is also set to zero, as displayed below or in (6):

$$ \begin{array}{*{20}{c}}  {0={{P}_{{{{S}_{{37}}}}}}=-\frac{1}{5}\left( {{{P}_{{{{S}_{{36}}}}}}{{r}_{{S,all,1}}}+{{P}_{{{{S}_{{35}}}}}}{{r}_{{S,all,2}}}+{{P}_{{{{S}_{{34}}}}}}{{r}_{{S,all,3}}}+{{P}_{{{{S}_{{33}}}}}}{{r}_{{S,all,4}}}+{{P}_{{{{S}_{{32}}}}}}{{r}_{{S,all,5}}}} \right)} \\  {0={{P}_{{{{S}_{{38}}}}}}=-\frac{1}{6}\left( {{{P}_{{{{S}_{{37}}}}}}{{r}_{{S,all,1}}}+{{P}_{{{{S}_{{36}}}}}}{{r}_{{S,all,2}}}+{{P}_{{{{S}_{{35}}}}}}{{r}_{{S,all,3}}}+{{P}_{{{{S}_{{34}}}}}}{{r}_{{S,all,4}}}+{{P}_{{{{S}_{{33}}}}}}{{r}_{{S,all,5}}}+{{P}_{{{{S}_{{32}}}}}}{{r}_{{S,all,6}}}} \right)} \\  \vdots  \\  \end{array} $$

More generally, we may extend the formulae for the non-existing sulphur isotopes ${P}_{S_{32+i}}$ with (*i* ≥ 5) as a function of the powered sulphur roots *r*_*S*_^− *i*^ and only the stable isotopes with non-zero probabilities as:

6 $$ 0={P}_{S_{32+i}}=-\frac{1}{i}\left({P}_{S_{36}}{r}_{S, all,\left(i-4\right)}+{P}_{S_{34}}{r}_{S, all,\left(i-2\right)}+{P}_{S_{33}}{r}_{S, all,\left(i-1\right)}+{P}_{S_{32}}{r}_{S, all,i}\right) $$

In turn, the previous equation can be represented as a calculation to obtain the powered sulphur roots:

7 $$ {r}_{S, all,i}=-{\left({P}_{S_{32}}\right)}^{-1}\left({P}_{S_{36}}{r}_{S, all,\left(i-4\right)}+{P}_{S_{34}}{r}_{S, all,\left(i-2\right)}+{P}_{S_{33}}{r}_{S, all,\left(i-1\right)}\right) $$

It should be noted that Equation (7) is a simple linear equation that can be calculated simultaneously with the iterative BRAIN procedure. Indeed, we do not have to calculate roots of polynomial *Q*_*S*_(*I*) anymore. The root omitting procedure can be combined with the recurrence of constant length [RCL] method to keep the computational requirements constant in time as discussed in the Results and Discussion section.

## Results and Discussion

The BRAIN method has already been extensively compared with other methods for isotope distribution calculation [4, 6, 7, 9]. For this reason, we will restrict the evaluation of BRAIN 2.0 to the original BRAIN method. To keep the comparison as transparent as possible, we have disabled the computation of center-masses in the original BRAIN method because BRAIN 2.0 cannot calculate this metric. As the presented improvements are mainly useful for large molecules, we restrict the comparison to the four heavy biomolecules displayed in Supplementary Table S1. For small molecules (e.g., peptides), the original BRAIN is better suited because the interest is mainly on the lighter isotope variants. Moreover, for light molecules, the isotope distribution contains too few isotope variants to enable the [RCL] option safely (i.e., arrive at the point that previous calculations of *ψ*_*l*_ becomes ignorable). Furthermore, it should be noted that [RCL], [LSP], and [RO] are three innovations that can be implemented independently from each other. Since the focus of the evaluation is on the computational speed and accuracy of the calculated isotope distribution between BRAIN [27] and BRAIN 2.0, we only include [RCL] and [LSP] in the comparison. The root omitting procedure for all elements in the periodic table is implemented in the original BRAIN method in C++ and is available at https://code.google.com/p/brain-isotopic-distribution/. Root omitting [RO] has no impact on the asymptotic algorithmic efficiency as stated by Hu et al. [25], but represent molecules by generic equations that allow calculation of the roots in a recursive manner without numerical root-finding. The Bioconductor package in R does not include the root omitting option as its current version mainly serves the calculation of peptides and proteins that only allow C, H, N, O, and S atoms.

The accuracy of the [RCL] and [LSP] implementation is assessed by comparing the relative isotope distributions from BRAIN and BRAIN 2.0. For this purpose, the Pearson *χ* ^2^ error statistic on the consecutive isotope ratios is calculated that provides a measure for the similarity of the generated isotope distributions:

8 $$ {\chi}^2={\displaystyle \sum_{i={n}_{start}}^{n_{stop}}}\;\frac{{\left({R}_i^I-{R}_i^{II}\right)}^2}{R_i^I} $$

with *R*_*i*_^*I*^ and *R*_*i*_^*II*^ being the ratios between the probabilities of consecutive isotope variants (i.e., ${\scriptscriptstyle \frac{q_{i+1}}{q_i}}$), of the returned isotope distribution from BRAIN and BRAIN 2.0, respectively.

In a first assessment, the methods are compared with only the [RCL] option implemented. The stopping peak for a given molecule is specified by the heuristic in the BRAIN paper [9]:

9 $$ {n}_{\mathrm{stop}}= max\left(2\times \left\lceil {\mathrm{mass}}_{\mathrm{average}}-{\mathrm{mass}}_{\mathrm{lightestIsotopeVariant}}\right\rceil, 5\right), $$

where ⌈and⌉ are the ceiling corner brackets that indicate the integer ceiling function (i.e.,the nearest integer not smaller than the value between the brackets). Recall that in this comparison the starting peak is equal to the lightest isotope variant, i.e., *n*_start_ = 1 as required by BRAIN. The constant memory *d* for [RCL] was selected according to the following rule of thumb:

10 $$ \left\lceil lo{g}_{10}(M)+5\right\rceil $$

where *M* is the mass of the lightest isotope variant of the molecule. In other words, the parameter *d* is set to five plus the number of digits in the integer part of lightest isotope mass. For each molecule, the elapsed system times is measured and divided by the number of times the calculation is repeated. To obtain a stable estimate for the timing, we perform 100 independent calculations (the performance tests presented in this manuscript were made on a machine with two Intel(R) Core(TM)2 CPU 6600@2.40GHz). The obtained results for the selected molecules are displayed in Supplementary Table S1. It can be observed that the agreement between BRAIN and BRAIN 2.0 is large in term of the obtained isotope distributions, as the *χ* ^2^ error statistic is very small. Indeed, reducing the memory to constant length and ignoring previous states in the recursion does not affect the accuracy of the computed isotope distribution. However, the [RCL] option reduces the asymptotic complexity of the algorithm because only a memory of size *O(d)* is needed. Hence, [RCL] yields an improvement in speed, as can be noted from the three last columns of Supplementary Table S1.

In a second assessment, we compare BRAIN with BRAIN 2.0 when only the [LSP] option is activated. The burn-in period *b* for [LSP] is also defined by the rule of thumb in Equation (10). Note that the core of the algorithm in both BRAIN and BRAIN 2.0 is unchanged by [LSP] since the summation is not restricted to a constant memory. However, because we represent the isotope distribution as relative intensities in BRAIN 2.0, we can avoid the prerequisite to start from the lightest isotope variant. Such an approach is not possible in the original BRAIN method and requires the heuristic in Equation (9), which leads to a calculation of too many isotope variants. It is obvious that fewer peaks (i.e., iterations), lead to a speed-up of the calculation. A complete discussion on the use of heuristics and their impact on algorithmic efficiency is provided by Hu et al. [25] and Fernandez-de-Cossio Diaz and Fernandez-de-Cossio [6]. Since the [LSP] option enables a more efficient heuristic to define the starting and stopping peak for a given molecule, we rely on the heuristic described by Rockwood et al. [17] and used by Fernandez-de-Cossio Diaz and Fernandez-de-Cossio [6]:

11 $$ N=\left\lceil \alpha \sqrt{\left(1+{\sigma}^2\right)}\right\rceil, $$

where *α* is set to 10 and *σ* is the standard deviation of the mass distribution. Note that a smaller value for *α* is used than the value specified in [6] as it gives smaller intervals with still very high coverage of the isotope distribution. The number of peaks *N* is centered on the average mass of the particular molecule. A larger *α* value will result in a wider span of the isotope distribution and a more complete coverage, but a longer computing time, as more peaks are included in the calculation. It should be noted that for a molecule, BRAIN and BRAIN 2.0 with [LSP] return an isotope distribution that contains a different number of peaks. The similarity between the returned distributions is evaluated on the peaks that are mutually present. As stated by Hu et al. [25], the heuristic in Equation (9) includes the range specified by Equation (11). The result for BRAIN 2.0 with the [LSP] option is given in Supplementary Table S2. Interestingly, the number of peaks can be reduced tremendously without affecting the coverage of the isotope distribution, which is over 99.999*%* in all cases as calculated by BRAIN. This result indicates that for very large molecules, the original BRAIN method calculates isotope peaks with a very low and ignorable probability, leading to a suboptimal use of computation time.

The criterion used to define the burn-in period *b* is sufficient, since the returned distributions have a good agreement as illustrated by the small values for the Pearson *χ* ^2^ error statistic. For the molecules presented in Supplementary Table S2, at most 11 burn-in steps are required, which indicate that the relative isotope intensities converge quickly to the actual isotope ratios.

In the third assessment, both [RCL] and [LSP] will be activated and compared with the original BRAIN method. The burn-in *b* for [LSP] and the constant memory *d* for [RCL] are set according to Equation (10). Doing so, the parameters *b* and *d* are set equally. It should be underlined that this heuristic is simplistic; in particular, the parameters may be set independently from each other. The results are displayed in Table 1; they indicate that while there is a big gain in time (last three columns), only tiny differences are observed in the accuracy of the isotope distribution (column ‘*χ* ^2^’). For *r* ratios, or equivalently *b* + *r* + 1 peaks, a constant memory of length *d* leads to a time complexity *C*_*t*_ = *O*(*d* × (*r* + *b* + 1)) and a memory complexity *C*_*m*_ = *O*(*d* + *r*). If heuristic formula (10) is applied for both [RCL] and [LSP], then an asymptotic complexity of *C*_*t*_ = *O*(*log*(*M*)(*r* + *log*(*M*))) and *C*_*m*_ = *O*(*log*(*M*) + *r*) is obtained as a function of the molecular mass *M*.

**Table 1:** [RCL] and [LSP] Improvements Tested for 4 Heavy Biomolecules from [26]. Speed is Measured as Elapsed Time in Seconds and Averaged from 100 Independent runs. For this comparison, we used heuristic from [9] (cf. Equation (9)) for original BRAIN and heuristic from [6] (cf. Equation (11, *α* = 10)) for BRAIN 2.0 with both [RCL] and [LSP] improvements. Center-masses calculations are disabled in both cases.

i.d.	*monoMass*(*Da*)	*b*	*d*	*χ* ^2^	*speed*_*BRAIN*_	*speed*_*BRAIN* 2_	Improvement
1	112824	11	11	2.39e-13	0.00873	0.00473	1.85
2	186387	11	11	9.79e-14	0.0138	0.0054	2.56
3	398470	11	11	5.02e-14	0.0336	0.007	4.8
4	533403	11	11	1.87e-14	0.0493	0.00766	6.43

It is obvious that the starting point of a calculation (i.e., *n*_start_ − *b*) cannot be smaller than the lightest isotope variant peak. The starting value of the algorithm should be at least equal to the lightest variant, as in the original BRAIN method. In contrast, [RCL] can always be applied on the condition that the returned number of peaks exceeds the constant memory *d*. In the case the iteration is started from the lightest isotope variant, exact values for the isotope probabilities are estimated with [RCL] disabled or enabled.

## Conclusions

We illustrate that the iterative algebraic approach used in the BRAIN algorithm for calculating the isotope distribution can be optimized to promote a more efficient use of memory and computation time. For this purpose, we propose two developments. First, the recurrence of constant length [RCL] will restrict the number of terms in the summations to a constant value. This development has an impact on the asymptotic complexity of the algorithm. The second development allows for a user-defined starting point [LSP], which enables more efficient heuristics to define the number of peaks returned by the algorithm. For example, the study of one particular isotope ratio (e.g., the ratio between the most abundant isotope peak and its consecutive peak) could be performed accurately by [LSP] and [RCL] switched on. Although the investigated peaks do not necessary cover a large part of the whole distribution, the ratio is estimated very accurately. This approach was not possible in the original BRAIN method, where the iterative calculation had to start from the lightest isotope variant. The implementation of a recurrence of constant length [RCL] and late starting point [LSP] will be added as an option to the existing Bioconductor BRAIN package [27] (http://www.bioconductor.org/packages/release/bioc/html/BRAIN.html). Root omitting [RO] enables an elegant and generic representation of elements and avoids the calculation of roots. However, the procedure for root omitting [RO] is not implemented in the Bioconductor BRAIN package as this version of the package is mainly intended to calculate isotope distributions for peptides and proteins. As mentioned earlier, root omitting is implemented in the C++ software available online for all the elements in the periodic table. We applied the proposed concepts on biomolecules that contain only five elements (i.e., C, H, N, O, S). These concepts can be easily extended to other elements as well; however, caution should be applied when porting these principle to other elements. The numerical properties explained in the recurrence of constant length can differ for other elements as they exhibit a different elemental isotope distribution. For instance, elements such as bromine or chlorine will converge at a slower rate to ignorable values for *ψ*_*l*_. Therefore, depending on the atomic composition of a molecule, the parameter that defines the length of the memory *d* may vary. Finally, the achieved improvements in computation time are substantial but seem ignorable for the user when looking at a single isotope calculation. Both BRAIN and BRAIN 2.0 are able to quickly calculate the isotope distribution. However, when the isotope distribution is required for large protein databases or BRAIN 2.0 is used to generate hypothetical isotope distributions in an optimization procedure, then the [RCL] and [LSP] improvements will be noticeable by the user.

## Electronic supplementary material

Below is the link to the electronic supplementary material.

Supplementary Table S1[RCL] improvement comparison tested for 4 heavy biomolecules from [26]. Speed is measured as elapsed time in seconds and averaged from 100 independent runs. The distribution coverage for investigatedpeak intervals is always very high (over 99.999 % according to BRAIN). For this assessment we used heuristic from [9] presented in Eq. **9** for both original BRAIN and BRAIN 2.0. with only [RCL] improvement. Center-masses calculations are disabled in both cases. The column *improvement* shows the ratio ${\scriptscriptstyle \frac{ spee{d}_{BRAIN}}{ spee{d}_{BRAIN2}}}$ (DOC 52 kb)

Supplementary Table S2 [LSP] improvement comparison tested for 4 heavy biomolecules from [26]. Speed is measured as elapsed time in seconds and averaged from 100 independent runs. The distribution coverage for investigated peak intervals is always very high (over 99.999 % according to BRAIN). For this comparison we used heuristic from [9] (cf. Eq. **9**) for original BRAIN and heuristic from [6] (cf. Equation 11, *α* = 10) for BRAIN 2.0. with only [LSP] improvement. No center-masses are calculated in both cases to make a comparison fair. For BRAIN 2.0 the total number of estimated peaks equals to *N* + *b*, i.e. it includes also the burn-in period. We observe a trend that favors BRAIN 2.0 with [LSP] improvement. (DOC 50 kb)

Supplementary Figure S1 We calculated BRAIN 2.0 with [RCL] and [LSP] enabled for four heavy biomolecules (cf. Supplementary Table S1) with heuristic from [6] (cf. Eq. 11, *α* = 10). In Panel (A) the burn-in parameter *b* is set to 11 whilst the memory of the summation *d* is varied from 4 to 14 (x-axis). For each combination of parameters *b* and *d*, the returned isotope distribution is compared to the result of the original BRAIN method by means of the Pearson *χ* ^2^ error statistic. This error is presented in the y-axis as a logarithmic scale with base 10. Panel (B) is similar as Panel (A) except that *d* is kept fixed at 11 whilst *b* changes from 3 to 21 (x-axis). Both panels exhibit a decreasing trend for the Pearson *χ* ^2^ error statistic, indicating that more accurate results can be obtained when increasing the burn-in and the length of the summation at the cost of computation time. When *b* = *d* = 11 the error is smaller than 10^− 12^. On the other hand, when the parameters are chosen inappropriately, BRAIN 2.0 could potentially induce large errors in the isotope distribution. The heuristic in Eq. 10 (which equals 11 in case of the four analyzed molecules) is provided to safe-guard the users for such a misspecification. (PNG 23 kb)

(PNG 19 kb)

## References

[1] McIlwain S, Page D, Huttlin E, Sussman M (2008). Using dynamic programming to create isotopic distribution maps from mass spectra. Bioinformatics.

[2] Snider RK (2007). Efficient calculation of exact mass isotopic distributions. J. Am. Soc. Mass Spectrom..

[3] Li L, Kresh J, Karabacak M, Cobb J, Agar J, Hong P (2008). A hierarchical algorithm for calculating the isotopic fine structures of molecules. J. Am. Soc. Mass Spectrom..

[4] Böcker, S.: Comment on: "An Efficient Method to Calculate the Aggregated Isotopic Distribution and Exact Center-Masses" by Jürgen Claesen, Piotr Dittwald, Tomasz Burzykowski, Dirk Valkenborg. J. Am. Soc. Mass Spectrom. **23**, 753–763 (2012) and J. Am. Soc. for Mass Spectrom. **23**, 1826–1827 (2012) 10.1007/s13361-012-0402-2 22673835

[5] Claesen J, Dittwald P, Burzykowski T, Valkenborg D (2012). Reply to the comment on: *J*. Am. Soc. Mass Spectrom..

[6] Fernandez-de Cossio Diaz J, Fernandez-de Cossio J (2012). Computation of isotopic peak center-mass distribution by Fourier transform. Anal. Chem..

[7] Scheubert K, Hufsky F, Böcker S (2013). Computational mass spectrometry for small molecules. J. Chem. Inform..

[8] Valkenborg D, Mertens I, Lemière F, Witters E, Burzykowski T (2012). The isotopic distribution conundrum. Mass Spectrom. Rev..

[9] Claesen J, Dittwald P, Burzykowski T, Valkenborg D (2012). An efficient method to calculate the aggregated isotopic distribution and exact center-masses. J. Am. Soc. Mass Spectrom..

[10] Yamamoto H, McCloskey JA (1977). Calculations of isotopic distribution in molecules extensively labeled with heavy isotopes. Anal. Chem..

[11] Brownawell M, Fillippo JS (1982). A program for the synthesis of mass spectral isotopic abundances. J. Chem. Educ..

[12] Olsen, C.E.: A pascal program for micro-computers for calculations of compositions and isotope clusters from accurate mass measurements. Int. J. Mass Spectrom. Ion Phys. **47**, 337–340 (1983)

[13] Hibbert, D.B.: A Prolog program for the calculation of isotope distributions in mass-spectrometry. Chem. Intell. Lab. Syst. **6**, 203–212 (1989)

[14] Pulfer JD, Derrick PJ (1991). Simulation of isotopic peak patterns for high-mass oligomers and polynuclidic transition-metal salts. Aus. J. Chem..

[15] Datta BP (1997). Polynomial method of molecular isotopic abundance calculations: a computational note. Rapid Commun. Mass Spectrom..

[16] Rockwood AL (1995). Relationship of Fourier transforms to isotope distribution calculations. Rapid Commun. Mass Spectrom..

[17] Rockwood AL, Van Orden SL, Smith RD (1995). Rapid calculation of isotope distributions. Anal. Chem..

[18] Rockwood AL, Van Orden SL, Smith RD (1996). Ultrahigh resolution isotope distribution calculations. Rapid Commun. Mass Spectrom..

[19] Rockwood AL, Van Orden SL (1996). Ultrahigh-speed calculation of isotope distributions. Anal. Chem..

[20] Rockwood AL, Van Orman JR, Dearden DV (2004). Isotopic compositions and accurate masses of single isotopic peaks. J. Am. Soc. Mass Spectrom..

[21] Yergey JA (1983). A general approach to calculating isotopic distributions for mass spectrometry. Int. J. Mass Spectrom. Ion Phys..

[22] Yergey JA, Heller D, Hansen G, Cotter RJ, Fenselau C (1983). Isotopic distributions in mass spectra of large molecules. Anal. Chem..

[23] Rockwood AL, Haimi P (2006). Efficient calculation of accurate masses of isotopic peaks. J. Am. Soc. Mass Spectrom..

[24] Böcker, S., Letzel, M.C., Lipták, Z., Pervukhin, A.: Sirius: decomposing isotope patterns for metabolite identification. Bioinformatics **25**, 218–224 (2009) 10.1093/bioinformatics/btn603 PMC2639009 19015140

[25] Hu, H., Dittwald, P., Zaia, J., Valkenborg, D.: Comment on the computation of isotopic peak center-mass distribution by Fourier transform. Anal. Chem. **85**, 12189–12192 (2013) 10.1021/ac402731h PMC4119064 24187947

[26] Olson MT, Yergey AL (2009). Calculation of the isotope cluster for polypeptides by probability grouping. J. Am. Soc. Mass Spectrom..

[27] Dittwald P, Claesen J, Burzykowski T, Valkenborg D, Gambin A (2013). BRAIN: a universal tool for high-throughput calculations of the isotopic distribution for mass spectrometry. Anal. Chem..

