# Supplementary material for: BRAIN 2.0: Time and Memory Complexity Improvements in the Algorithm for Calculating the Isotope Distribution
Source: J Am Soc Mass Spectrom. 2014 Feb 12;25(4):588–94. doi: 10.1007/s13361-013-0796-5 (PMC3953541; doi:10.1007/s13361-013-0796-5)
Supplement: Supplementary file 1 — [RCL] improvement comparison tested for 4 heavy biomolecules from [26]. Speed is measured as elapsed time in seconds and averaged from 100 independent runs. The distribution coverage for investigatedpeak intervals is always very high (over 99.999 % according to BRAIN). For this assessment we used heuristic from [9] presented in Eq. 9 for both original BRAIN and BRAIN 2.0. with only [RCL] improvement. Center-masses calculations are disabled in both cases. The column improvement shows the ratio ${\scriptscriptstyle \frac{ spee{d}_{BRAIN}}{ spee{d}_{BRAIN2}}}$speedBRAINspeedBRAIN2 (DOC 52 kb) [file 13361_2013_796_MOESM1_ESM.doc]

# Supplementary materials

Supplementary Table S1: [RCL] improvement comparison tested for 4 heavy biomolecules from  [26]. Speed is measured as elapsed time in seconds and averaged from 100 independent runs. The distribution coverage for investigatedpeak intervals is always very high (over 99.999% according to BRAIN). For this assessment we used heuristic from   [9] presented in Equation 9 for both original BRAIN and BRAIN 2.0. with only [RCL] improvement. Center-masses calculations are disabled in both cases. The column *improvement* shows the ratio $\frac{speed_{BRAIN}}{speed_{BRAIN2}}$.

| *id* | *formula* | $monoMass(Da)$ | *d* | $n_{start}$ | $n_{stop}$ | *N* | $\chi^{2}$ | $speed_{BRAIN}$ | $speed_{BRAIN2}$ | *improvement* |
| --- | --- | --- | --- | --- | --- | --- | --- | --- | --- | --- |
| 1 | $C_{5047}H_{8014}N_{1338}O_{1495}S_{48}$ | 112824 | 11 | 1 | 143 | 143 | 1.07e-11 | 0.00863 | 0.00588 | 1.47 |
| 2 | $C_{8574}H_{13378}N_{2092}O_{2392}S_{77}$ | 186387 | 11 | 1 | 239 | 239 | 2.45e-11 | 0.0137 | 0.00873 | 1.57 |
| 3 | $C_{17600}H_{26474}N_{4752}O_{5486}S_{197}$ | 398470 | 11 | 1 | 506 | 506 | 7.65e-11 | 0.0336 | 0.0162 | 2.07 |
| 4 | $C_{23832}H_{37816}N_{6528}O_{7031}S_{170}$ | 533403 | 11 | 1 | 664 | 664 | 6.2e-11 | 0.0484 | 0.0208 | 2.33 |
