# Supplementary material for: BRAIN 2.0: Time and Memory Complexity Improvements in the Algorithm for Calculating the Isotope Distribution
Source: J Am Soc Mass Spectrom. 2014 Feb 12;25(4):588–94. doi: 10.1007/s13361-013-0796-5 (PMC3953541; doi:10.1007/s13361-013-0796-5)
Supplement: Supplementary file 2 — [LSP] improvement comparison tested for 4 heavy biomolecules from [26]. Speed is measured as elapsed time in seconds and averaged from 100 independent runs. The distribution coverage for investigated peak intervals is always very high (over 99.999 % according to BRAIN). For this comparison we used heuristic from [9] (cf. Eq. 9) for original BRAIN and heuristic from [6] (cf. Equation 11, α = 10) for BRAIN 2.0. with only [LSP] improvement. No center-masses are calculated in both cases to make a comparison fair. For BRAIN 2.0 the total number of estimated peaks equals to N + b, i.e. it includes also the burn-in period. We observe a trend that favors BRAIN 2.0 with [LSP] improvement. (DOC 50 kb) [file 13361_2013_796_MOESM2_ESM.doc]

Supplementary Table S2: [LSP] improvement comparison tested for 4 heavy biomolecules from  [26]. Speed is measured as elapsed time in seconds and averaged from 100 independent runs. The distribution coverage for investigated peak intervals is always very high (over 99.999% according to BRAIN). For this comparison we used heuristic from  [9] (cf. Equation 9) for original BRAIN and heuristic from  [6] (cf. Equation efform:ISOTOPICAheuristic, $\alpha=10$) for BRAIN 2.0. with only [LSP] improvement. No center-masses are calculated in both cases to make comparison fair. For BRAIN 2.0 the total number of estimated peaks equals to $N+b$, i.e. includes also burn-in period. We observe the trend favoring BRAIN 2.0 with [LSP] improvement.

|  | | | BRAIN | | | BRAIN 2.0 | | |  | | | |
| --- | --- | --- | --- | --- | --- | --- | --- | --- | --- | --- | --- | --- |
| *id* | $monoMass(Da)$ | *b* | $n_{start}$ | $n_{stop}$ | *N* | $n_{start}$ | $n_{stop}$ | *N* | $\chi^{2}$ | $speed_{BRAIN}$ | $speed_{BRAIN2}$ | *improvement* |
| 1 | 112824 | 11 | 1 | 143 | 143 | 26 | 117 | 92 | 1.75e-30 | 0.00859 | 0.00748 | 1.15 |
| 2 | 186387 | 11 | 1 | 239 | 239 | 61 | 178 | 118 | 8.63e-29 | 0.0136 | 0.00996 | 1.37 |
| 3 | 398470 | 11 | 1 | 506 | 506 | 167 | 338 | 172 | 2.08e-25 | 0.0336 | 0.0158 | 2.13 |
| 4 | 533403 | 11 | 1 | 664 | 664 | 235 | 429 | 195 | 8.68e-25 | 0.0484 | 0.0188 | 2.57 |
