# Supplementary figures and images for: BRAIN 2.0: Time and Memory Complexity Improvements in the Algorithm for Calculating the Isotope Distribution
Source: J Am Soc Mass Spectrom. 2014 Feb 12;25(4):588–94. doi: 10.1007/s13361-013-0796-5 (PMC3953541; doi:10.1007/s13361-013-0796-5)

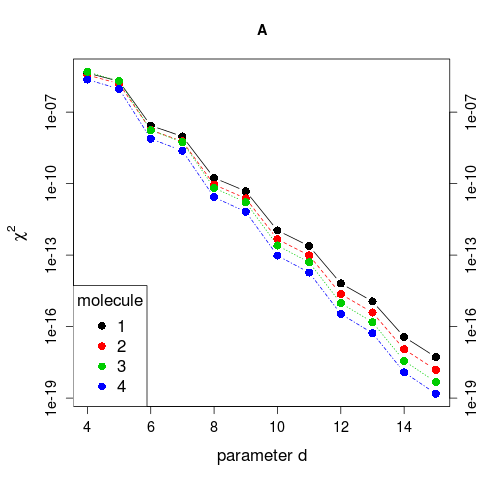

Supplement: Supplementary file 3 — We calculated BRAIN 2.0 with [RCL] and [LSP] enabled for four heavy biomolecules (cf. Supplementary Table S1) with heuristic from [6] (cf. Eq. 11, α = 10). In Panel (A) the burn-in parameter b is set to 11 whilst the memory of the summation d is varied from 4 to 14 (x-axis). For each combination of parameters b and d, the returned isotope distribution is compared to the result of the original BRAIN method by means of the Pearson χ 2 error statistic. This error is presented in the y-axis as a logarithmic scale with base 10. Panel (B) is similar as Panel (A) except that d is kept fixed at 11 whilst b changes from 3 to 21 (x-axis). Both panels exhibit a decreasing trend for the Pearson χ 2 error statistic, indicating that more accurate results can be obtained when increasing the burn-in and the length of the summation at the cost of computation time. When b = d = 11 the error is smaller than 10− 12. On the other hand, when the parameters are chosen inappropriately, BRAIN 2.0 could potentially induce large errors in the isotope distribution. The heuristic in Eq. 10 (which equals 11 in case of the four analyzed molecules) is provided to safe-guard the users for such a misspecification. (PNG 23 kb) [file 13361_2013_796_MOESM3_ESM.png]

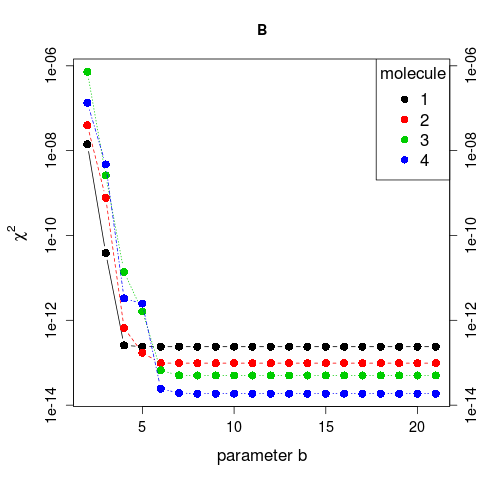

Supplement: Supplementary file 4 — (PNG 19 kb) [file 13361_2013_796_MOESM4_ESM.png]
